# Androgen receptor inhibitor ameliorates pulmonary arterial hypertension by enhancing the apoptosis level through suppressing the Notch3/Hes5 pathway

**DOI:** 10.3389/fphar.2025.1572489

**Published:** 2025-04-28

**Authors:** Jiayan Sun, Jiancheng Lin, Di Yin, Zetao Pan, Yuheng Ye, Yi Wang, Xiaowan Wang, Qiang Guo

**Affiliations:** ^1^ Medical College of Soochow Universuty, Soochow University, Suzhou, Jiangsu, China; ^2^ Medical Center of Soochow University, Soochow University, Suzhou, Jiangsu, China; ^3^ Department of Emergency and Critical Care Medicine, The Fourth Affiliated Hospital of Soochow University (Suzhou Dushu Lake Hospital), Suzhou, Jiangsu, China; ^4^ The First Affiliated Hospital of Soochow University, Suzhou, Jiangsu, China

**Keywords:** pulmonary arterial hypertension, androgen receptor, Notch3, Hes5, apoptosis

## Abstract

**Background:**

Pulmonary arterial hypertension (PAH) exhibits significant gender differences in prognosis, with male patients typically showing worse outcomes than females. These disparities may stem from differences in androgen receptor expression and activity. Clinical studies suggest that the androgen receptor plays a crucial role in the pathophysiology of PAH, influencing disease progression and treatment response. Despite the lack of targeted therapies for PAH, these findings have spurred investigations into the potential therapeutic role of androgen receptors. This study explores the role of androgen receptors in PAH and evaluates their therapeutic potential.

**Methods:**

PAH was induced in rats via intraperitoneal injection of monocrotaline (MCT). Following model establishment, Enzalutamide was administered every 3 days at 10 mg/kg once for a total of 7 times (21 days). A mouse model of PAH was developed by subcutaneously injecting SU5416 and exposing the mice to hypoxia. Androgen receptor knockout (AR^−/−^) mice were also utilized to investigate the role of androgen receptors in disease progression. Key indicators were compared across groups. The *in vivo* mechanisms through which androgen receptors influence PAH were examined in both rat and mouse models. Additionally, mouse pulmonary artery endothelial cells (PAECs) were cultured under hypoxic conditions to create an *in vitro* model of PAH, facilitating further investigation into the role of androgen receptors in disease pathogenesis.

**Results:**

Compared to the normal group, the model group exhibited significantly increased androgen receptor expression in rats, mice, and mPAECs. This was accompanied by pronounced pulmonary artery wall thickening, right ventricular hypertrophy, pulmonary fibrosis, elevated pulmonary artery pressure, and a reduced level of apoptosis both *in vivo* and *in vitro*. Furthermore, activation of the Notch3/Hes5 signaling pathway was observed. However, treatment with androgen receptor inhibitors or gene knockout significantly ameliorated these pathological changes. Apoptosis levels increased both *in vivo* and *in vitro*, and the activation of the Notch3/Hes5 signaling pathway was effectively inhibited.

**Conclusion:**

Our findings suggest that in both animal models and the hypoxic mPAECs, inhibition of androgen receptor expression leads to increased apoptosis via suppression of the Notch3/Hes5 signaling pathway. This mechanism likely contributes to the therapeutic effects observed, providing insights for potential treatment strategies targeting androgen receptors in pulmonary arterial hypertension.

## 1 Introduction

Pulmonary arterial hypertension (PAH) is a progressive disease characterized by the remodeling of pulmonary arterioles and right ventricular dysfunction. Clinical symptoms include dyspnea, fatigue, chest pain, and syncope. In severe cases, PAH can lead to right heart failure and death. Although PAH is considered a rare disease, its incidence is estimated at 15 to 50 cases per million people worldwide (Collaborators, 2025). Epidemiologically, female patients tend to have a longer survival period, while male patients often experience a more severe disease course and higher mortality rates. The primary pathological mechanisms of PAH involve endothelial cell dysfunction, smooth muscle cell proliferation, inflammatory responses, and mitochondrial metabolic disorders, which contribute to the narrowing and stiffening of small pulmonary arteries (Zhou et al., 2024). These mechanisms are closely linked to the regulation of apoptosis.

Gender differences in PAH are notable (Takase et al., 2021; Wits et al., 2023). The incidence is higher in females, while the prognosis tends to be worse in males. This discrepancy may be partly attributed to the influence of sex hormones, particularly androgens and their receptors. The androgen receptor (AR) not only regulates gender-specific gene expression but may also contribute to PAH pathogenesis by modulating apoptotic pathways (Godfrey et al., 2010; Lin et al., 2006; Wu et al., 2022; Lin et al., 2001; Zeng et al., 2023; Shibata et al., 2018; Kim et al., 2023).

Apoptosis is a key process in PAH pathogenesis (Jurasz et al., 2010; Li B. et al., 2023; Courboulin et al., 2012; Cao et al., 2020; Habbout et al., 2021; McMurtry et al., 2005). Under normal conditions, programmed cell death (PCD) maintains tissue homeostasis by regulating cell morphology and function. The Bcl-2 and Caspase3 families play crucial roles in apoptosis. However, during PAH development, pulmonary artery endothelial and smooth muscle cells show increased resistance to apoptosis, leading to abnormal cell proliferation and vascular remodeling (Yu et al., 2011; Liu et al., 2023; Gong et al., 2021; Korshunov and Berk, 2008; Sakao et al., 2009).

The Notch3/Hes5 signaling pathway regulates cell proliferation and apoptosis and is critical for maintaining endothelial and smooth muscle cell function, as well as blood vessel structural and functional stability (Ashino et al., 2016). The Notch3/Hes5 signaling pathway exhibits pro-proliferative and anti-apoptotic effects in the initiation and progression of various tumors, thereby promoting tumor growth (Akil et al., 2021; Haiaty et al., 2020; Palano et al., 2020; Qian et al., 2023; Li X. et al., 2023; Zhang et al., 2021). Additionally, this pathway plays a crucial role in vascular repair following injury. Therefore, its potential involvement in the pathogenesis of pulmonary hypertension warrants further investigation (Zhang et al., 2022; Dabral et al., 2016; Bodas et al., 2022).

This study uses rat, mouse, and mouse pulmonary artery endothelial cell models of PAH to investigate the role of androgen receptors in disease development and progression, as well as the underlying molecular mechanisms. Both the rat model treated with androgen receptor inhibitors and the androgen receptor knockout (AR^−/−^) mouse model exhibited increased apoptosis and downregulation of the Notch3/Hes5 signaling pathway. These findings were further confirmed by *in vitro* experiments using pulmonary artery endothelial cells. Based on these results, we propose that androgen receptor inhibition may exert therapeutic effects by suppressing the Notch3/Hes5 signaling pathway, leading to enhanced apoptosis of pulmonary artery endothelial cells. This apoptotic enhancement could counteract excessive endothelial cell proliferation, potentially alleviating the onset and progression of PAH (Huang et al., 2010).

## 2 Methods and materials

### 2.1 Experimental animals

All wild-type animals were obtained from Hangzhou Qizhen Laboratory Animal Co., Ltd. (Hangzhou, China). Male Sprague-Dawley rats, aged 8–9 weeks (approximately 250 g), and male C57BL/6J mice, aged 6–8 weeks (20–25 g), were used in this study (n = 6 in each group). Androgen receptor knockout (AR^−/−^) mice were supplied by GemPharmatech Co., Ltd. (Jiangsu, China). Upon arrival, the animals were acclimatized to standard housing conditions for 1 week, with access to a standard diet. Housing conditions included a 12-hour light/dark cycle, an ambient temperature of 23°C–25°C, and humidity levels of 50%–60%. A monocrotaline (MCT)-induced pulmonary hypertension model was established in rats via a single subcutaneous injection of 60 mg/kg MCT (MCE, China). Rats in the control group received an equivalent volume of saline. To establish the SU5416/hypoxia (SuHx) model, male C57BL/6J mice and AR^−/−^ mice, aged 6–8 weeks (20–25 g), were injected subcutaneously with SU5416 (20 mg/kg, MCE, China) once per week. Following each injection, mice were exposed to a hypoxic environment (10% O_2_) for 4 weeks. Control mice received equivalent saline injections and were housed in normoxic conditions. In the animal experiments conducted in this study, isoflurane was used for anesthesia. Anesthesia was induced in both rats and mice with isoflurane at concentrations of 3%–5%, and maintained at 1%–3%. Euthanasia was performed using carbon dioxide, which was introduced at a flow rate of 10% of the container volume per minute. The concentration of carbon dioxide in the chamber was gradually increased to over 70% to ensure rapid, humane euthanasia without causing distress to the animals.

### 2.2 Cell culture

Mouse pulmonary artery endothelial cells (PAECs) were obtained from FuHeng Biology (Shanghai, China) and cultured in endothelial cell-specific medium. Cells were divided into four experimental groups (n = 3): (1) Control (Con) group: Cells were maintained under normoxic conditions in a standard incubator at 37°C with 5% CO_2_. (2) Hypoxia (Hypoxia) group: Cells were cultured under hypoxic conditions (1% O_2_, 5% CO_2_) at 37°C to induce hypoxia. (3) Hypoxia and Enzalutamide (Hyp + En) group: Hypoxia-exposed cells were treated with Enzalutamide (MCE, China) at concentrations of 5 μM, 10 μM, 20 μM, and 40 μM to assess its effects. (4) Hypoxia, Enzalutamide, and TFA (Hyp + En + TFA) group: Hypoxia-exposed cells treated with Enzalutamide were subsequently treated with 50 μM of TFA (MCE, China), an agonist of the Notch3/Hes5 signaling pathway.

### 2.3 Measurement of right ventricular systolic pressure

Following the completion of the modeling procedure, mice were anesthetized with 1% isoflurane in oxygen. Right ventricular systolic pressure (RVSP) was measured using a Millar catheter (SPR-839), which was carefully inserted into the right ventricle via the right external jugular vein. RVSP waveforms were continuously recorded, and the data were analyzed using the PowerLab system and LabChart software. After the procedure, all mice were humanely euthanized via cervical dislocation, in accordance with ethical guidelines.

### 2.4 Measurement of right ventricular hypertrophy index

Following the euthanasia of the animal, the heart was promptly excised while utmost efforts were made to preserve its integrity. Subsequently, the surrounding connective tissue and intraventricular blood clots were carefully dissected away. Subsequently, the dry weight of each tissue component was precisely measured using an electronic balance. The right ventricular hypertrophy index (RVHI) was calculated as the ratio of right ventricular weight (RV) to the combined weight of the left ventricle (LV) and interventricular septum (S), expressed as RV/(LV + S).

### 2.5 Immunofluorescence (IF)

The tissue immunofluorescence procedure includes the following steps: deparaffinization and rehydration of tissue sections, antigen retrieval via heat-induced epitope retrieval (HIER), quenching of endogenous peroxidase activity, and blocking of non-specific binding. Sections were incubated with a primary antibody, followed by a fluorescent secondary antibody. Antibody-antigen complexes were visualized using a fluorescence microscope, and sections were mounted with an anti-fade reagent. For cell migration immunofluorescence, cells were cultured on coverslips, fixed with 4% paraformaldehyde, and permeabilized with Triton X-100. Non-specific binding was blocked, and cells were incubated with a primary antibody, followed by a fluorescent secondary antibody. After thorough washing to remove excess antibody, cells were mounted with an anti-fade reagent and observed under a fluorescence microscope.

### 2.6 Hematoxylin-eosin staining experiment

After tissue specimens were fixed and embedded in paraffin, they were sectioned into 5–7 μm slices using a microtome. The sections were then subjected to dewaxing and rehydration before staining. The tissue sections were stained with hematoxylin for 3–8 min, rinsed with running water, and differentiated in a 1% hydrochloric acid-alcohol solution for a few seconds. The sections were then rinsed again with running water, treated with 0.6% ammonia water to “blue” the tissue, and rinsed again. Next, the sections were stained with eosin for 1–3 min. Finally, the stained sections were dehydrated through a graded alcohol series and mounted with a suitable mounting medium for microscopic examination.

### 2.7 Immunohistochemistry (IHC)

Paraffin-embedded tissue sections were deparaffinized and rehydrated, followed by antigen retrieval to unmask epitope sites. Endogenous peroxidase activity was quenched using an appropriate blocking agent, and non-specific binding was blocked at room temperature for 1–1.5 h. The sections were then incubated with a primary antibody, followed by incubation with a secondary antibody conjugated to a detectable label. The antigen-antibody complex was visualized using DAB chromogenic development. Finally, the sections were examined under a microscope to assess the localization, distribution, and expression intensity of the target antigen.

### 2.8 Masson’s trichrome staining

After dewaxing and rehydrating the tissue sections through xylene and graded ethanol, they were stained with hematoxylin for 5–10 min to achieve a bluish-purple coloration, ensuring distinct staining of the cell nuclei. Following hematoxylin staining, the sections were briefly differentiated in a 1% hydrochloric acid-ethanol solution to remove excess stain, then rinsed thoroughly with running water to stabilize the staining. The sections were then immersed in ponceau-acid fuchsin solution for 5–10 min to stain cytoplasmic and muscle components, without rinsing afterward. To remove excessive red staining from non-collagen fibers, the sections were treated with phosphomolybdic acid solution for 3–5 min. The sections were then stained with aniline blue solution to enhance the visualization of collagen fibers, creating a sharp contrast. Final differentiation was performed using 1% glacial acetic acid to refine the staining. The sections were dehydrated through an ascending series of ethanol concentrations, cleared with xylene, and mounted with a suitable mounting medium. Finally, the sections were examined under a light microscope for structural and histological evaluation.

### 2.9 Western blot analysis

Western blotting was performed as follows: Fresh lung tissue from animals was immediately frozen in liquid nitrogen for subsequent protein extraction. Tissue was dissected using sterile scissors and forceps, and tissue lysates were prepared at a ratio of 1 mL of lysis buffer per 0.1 g of tissue. After homogenization and centrifugation, the supernatant was collected, and protein concentration was determined using the BCA assay. The samples were then mixed with loading buffer and heated at 100°C before being stored at −80°C. Proteins were separated using an appropriate pre-cast gel (GenScript, China) based on molecular weight, with sample volume determined by protein concentration. Electrophoresis was performed using MOPS running buffer (GenScript, China), followed by protein transfer to a PVDF membrane (Millipore, IPVH00010) using rapid transfer buffer (Beyotime, China). After transfer, the membrane was blocked with blocking buffer (Beyotime) and incubated overnight at 4°C with the primary antibody. The secondary antibody was incubated at room temperature for 1 h, and chemiluminescent detection was performed. Membranes were washed with 1× TBST (Solabio) between each step. All the antibodies used in Western blot were provided by Proteintech (Wuhan, China).

### 2.10 Cell scratch migration assay

The cell scratch assay was performed as follows: Horizontal lines were drawn on the bottom of a six-well plate using a marker. Cells were seeded into the plate and allowed to adhere. Once cells had adhered, a sterile 1 mL pipette tip was used to make a uniform scratch perpendicular to the marked lines, applying even pressure to create a wound area. The culture medium was replaced with serum-free medium, and drugs were added at various concentrations for the experimental groups. The initial scratch area was photographed under a microscope at 0 h. The plate was then incubated under standard culture conditions. After 24 h, images of the wound area were captured again to assess the rate of closure and cell migration.

### 2.11 Statistical analyses

Differences between two groups were assessed using the Welch’s t-test for independent samples. For comparisons across multiple groups, a one-way analysis of variance (ANOVA) followed by post-hoc analysis was performed. Data from at least six independent experiments were analyzed using GraphPad Prism version 9.5 software and are expressed as the mean ± standard deviation (SD). A p-value of less than 0.05 was considered statistically significant.

## 3 Results

### 3.1 AR is upregulated in lung tissues of MCT-treated rats, SU5416/hypoxia-treated mice, and PAECs cultured under hypoxic conditions

**FIGURE 1:**
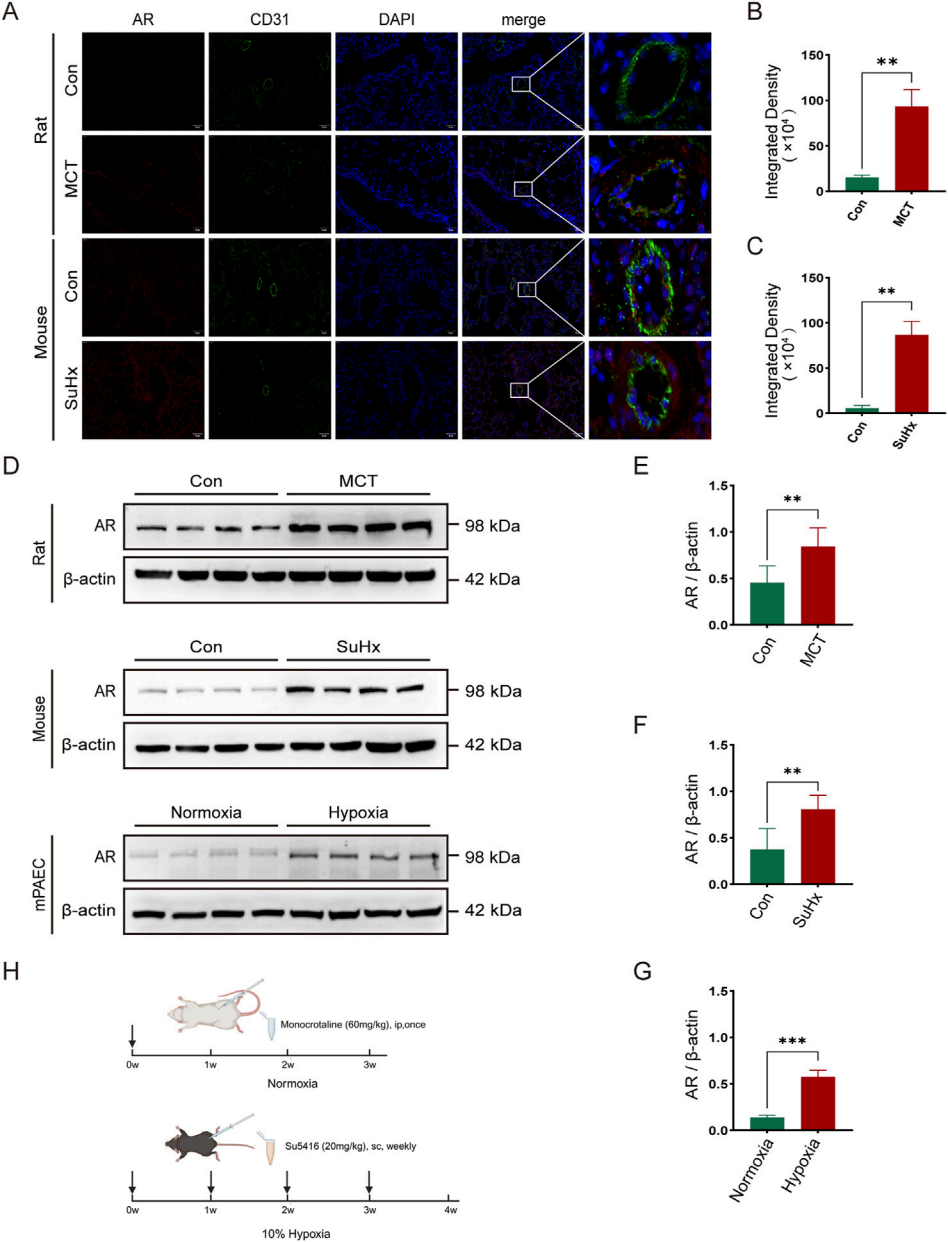
The expression of androgen receptors (AR) is elevated in the lung tissues of rats and mice and in the pulmonary artery endothelial cells of mice in pulmonary hypertension models. **(A)** Immunofluorescence of AR around arterial endothelial cells in the lung tissues of rats and mice; **(B)** Statistics of AR immunofluorescence intensity in rats; **(C)** Statistics of AR immunofluorescence intensity in mice; **(D)** Western blot of AR expression in various models; **(E)** Quantitative analysis of AR in rat lung tissues, **(F)** in mouse lung tissues, and **(G)** in mouse pulmonary artery endothelial cells; **(H)** Timeline of establishing pulmonary hypertension models in rats and mice (created in https://BioRender.com). **p < 0.01, ***p < 0.001.

To investigate androgen receptor (AR) expression in pulmonary arterial hypertension (PAH), we established a rat model by administering a single intraperitoneal injection of monocrotaline (MCT). For the mouse model, we subcutaneously injected SU5416 once a week and exposed the mice to a 10% hypoxic environment for 4 weeks (Figure 1H). To create an *in vitro* PAH model, mouse pulmonary artery endothelial cells (PAECs) were incubated under 1% hypoxic conditions. Immunofluorescence staining of lung sections from normal and model groups of rats and mice was performed to assess AR expression. Co-immunofluorescence staining using AR and the vascular endothelial cell marker CD31 revealed increased AR expression in the pulmonary artery endothelium of model rats and mice (Figures 1A–C). Western blot analysis of protein extracts from lung tissues and PAECs in the normal and model groups confirmed that AR expression levels were elevated in the model rats and mice compared to controls (Figures 1D–G).

### 3.2 Cardiopulmonary function is significantly improved in rats following enzalutamide treatment and in AR^−/−^ mice with PAH

**FIGURE 2:**
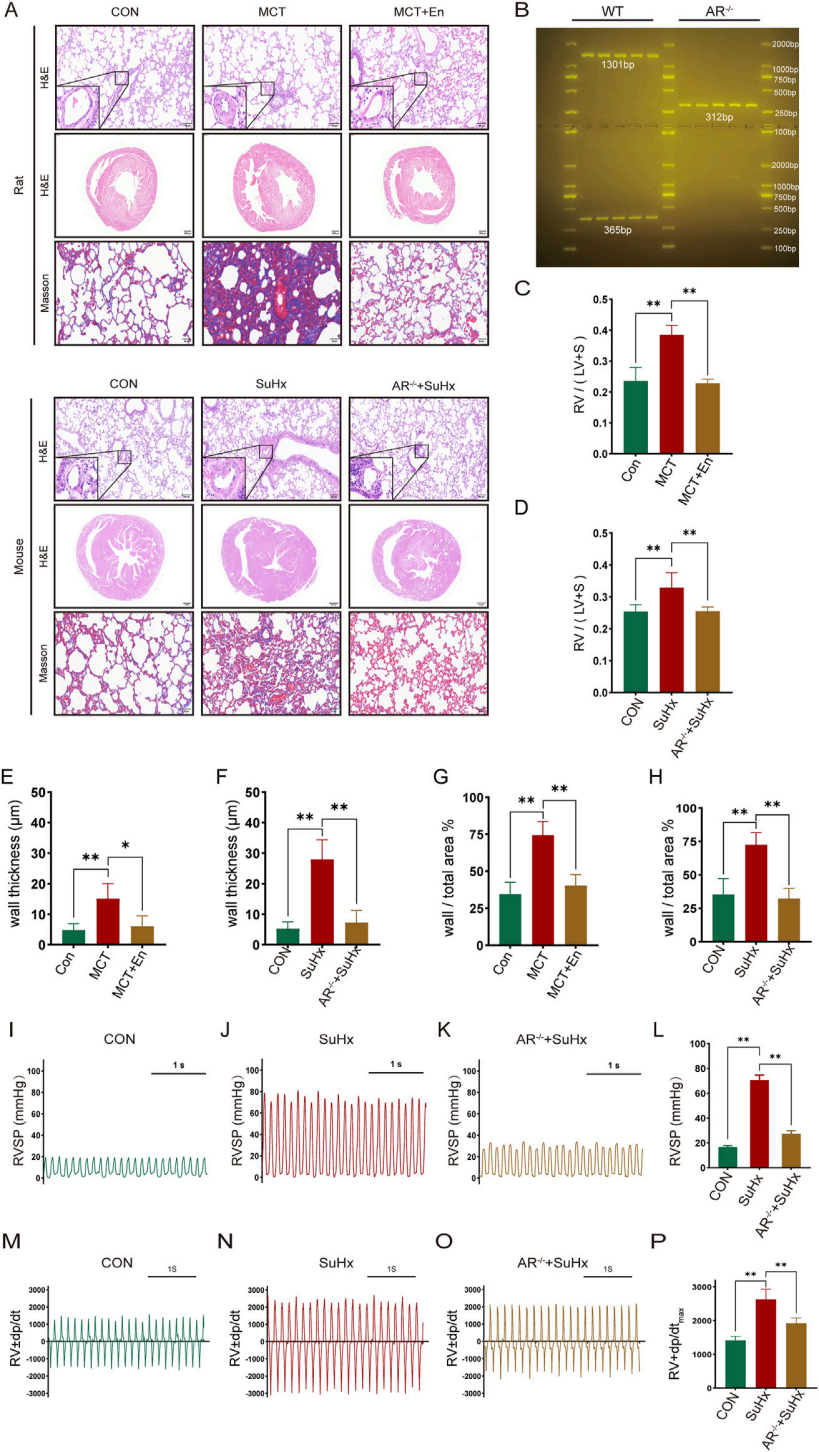
The cardiopulmonary function of rats treated with AR inhibitors and AR^−/−^ mice is improved compared with that of the model group. **(A)** HE staining of the heart and lung tissues and Masson staining of lung tissues; **(B)** Identification results of gel electrophoresis of AR^−/−^ mice; **(C)** Right ventricular hypertrophy index of rats; **(D)** Right ventricular hypertrophy index of mice; **(E)** Statistics of the wall thickness of small pulmonary arteries in rats and **(F)** in mice; **(G)** Ratio of the wall area to the whole arterial lumen area of small pulmonary arteries in rats and **(H)** in mice; **(I)** Right ventricular systolic pressure waveforms of normal mice, **(J)** model mice, and **(K)** AR^−/−^ mice, and **(L)** corresponding statistical graphs; **(M)** Rate of change of right ventricular systolic pressure of normal mice, **(N)** model mice, and **(O)** AR^−/−^ mice, and **(P)** statistical graphs of maximum values. *p < 0.05, **p < 0.01.

The study included three groups of rats (n = 6 in each group): the normal group, the MCT group, and the MCT + Enzalutamide (En) group. Similarly, mice were divided into the normal group, the SuHx group, and the SuHx + AR^−/−^ group (n = 6 in each group). AR^−/−^ male mice were generated by breeding heterozygous AR^−/−^ females with wild-type males, and genotyping was confirmed via DNA gel electrophoresis (Figure 2B). Compared to the normal group, MCT-treated rats and wild-type mice subjected to SU5416/hypoxia modeling exhibited significantly thickened arterial walls, right ventricular hypertrophy, and aggravated pulmonary fibrosis. However, in Enzalutamide-treated rats and AR^−/−^ mice, hematoxylin and eosin (HE) staining of lung tissues revealed improvements in pulmonary artery wall thickening, while HE staining of heart tissues indicated a reduction in right ventricular hypertrophy. Additionally, Masson staining of lung tissues demonstrated alleviation of pulmonary fibrosis, and the right ventricular hypertrophy index was significantly reduced (Figures 2A,C–H). Right heart catheterization using a Millar catheter was performed to measure right ventricular systolic pressure (RVSP), an indirect indicator of pulmonary artery pressure, and the maximum rate of pressure change in the right ventricle (dp/dt_max_), which reflects cardiac contractility. Wild-type mice exposed to SU5416/hypoxia showed significantly elevated RVSP and dp/dt_max_ compared to the normal group. In contrast, AR^−/−^ mice exhibited reduced RVSP and dp/dt_max_ compared to wild-type mice (Figures 2I–P).

### 3.3 Apoptosis levels are significantly increased in rats following enzalutamide treatment and in AR^−/−^ mice with PAH

**FIGURE 3:**
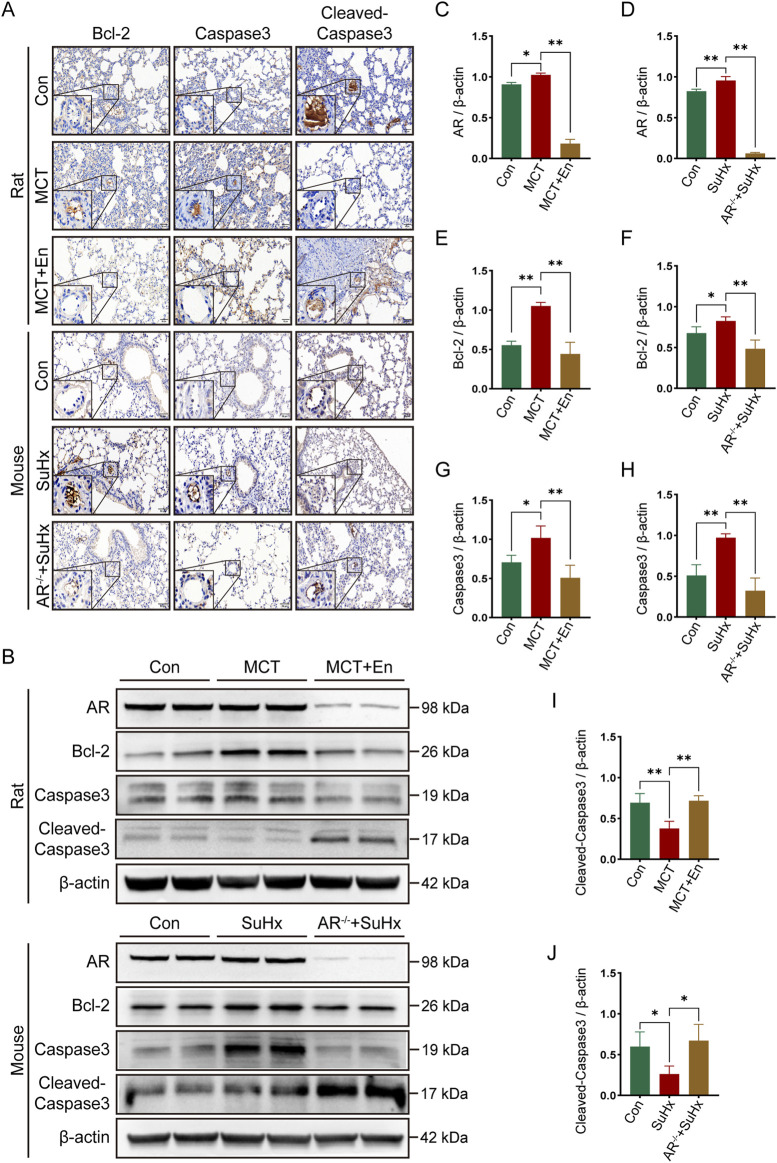
The apoptosis level is upregulated in the lung tissues of rats treated with AR inhibitors and AR^−/−^ mice. **(A)** Immunohistochemical staining of Bcl-2, Caspase3, and Cleaved-Caspase3 around pulmonary artery endothelial cells in the lung tissues of rats and mice; **(B)** Western blot detection in the lung tissues of rats and mice; Quantitative statistics of **(C)** AR, **(E)** Bcl-2, **(G)** Caspase3, and **(I)** Cleaved-Caspase3 in the lung tissues of three groups of rats; Quantitative statistics of **(D)** AR, **(F)** Bcl-2, **(H)** Caspase3, and **(J)** Cleaved-Caspase3 in the lung tissues of three groups of mice. *p < 0.05, **p < 0.01.

To evaluate apoptosis, we selected three common markers—Bcl-2, Caspase3, and Cleaved-Caspase3—and performed immunohistochemical staining and Western blot analysis on lung tissues from rats and mice in three experimental groups. Bcl-2, an anti-apoptotic protein, typically decreases with increasing apoptosis levels *in vivo*. Cleaved-Caspase3, the active form of Caspase3, increases with heightened apoptosis, while total Caspase3 levels, as a substrate for activation, tend to decline. Immunohistochemical analysis of lung sections revealed reduced apoptosis in the model groups, as indicated by higher expression of Bcl-2 and Caspase3. Conversely, apoptosis was elevated around pulmonary artery endothelial cells in MCT-treated rats receiving Enzalutamide and in SuHx mice with AR^−/−^ (Figure 3A). Proteins extracted from lung tissues were further analyzed by Western blotting. In the model groups, Bcl-2 and Caspase3 expression were elevated, whereas their expression decreased in the MCT + En and SuHx + AR^−/−^ groups. In contrast, Cleaved-Caspase3 expression, which was reduced in the model groups, was significantly increased in the MCT + En and SuHx + AR^−/−^ groups (Figures 3B–J). These results indicate that apoptosis levels were higher in the MCT + En rats and SuHx + AR^−/−^ mice compared to their respective model groups, consistent with the immunohistochemical findings.

### 3.4 The Notch3/Hes5 signaling pathway is inhibited in enzalutamide-treated rats and AR^−/−^ mice with PAH

**FIGURE 4:**
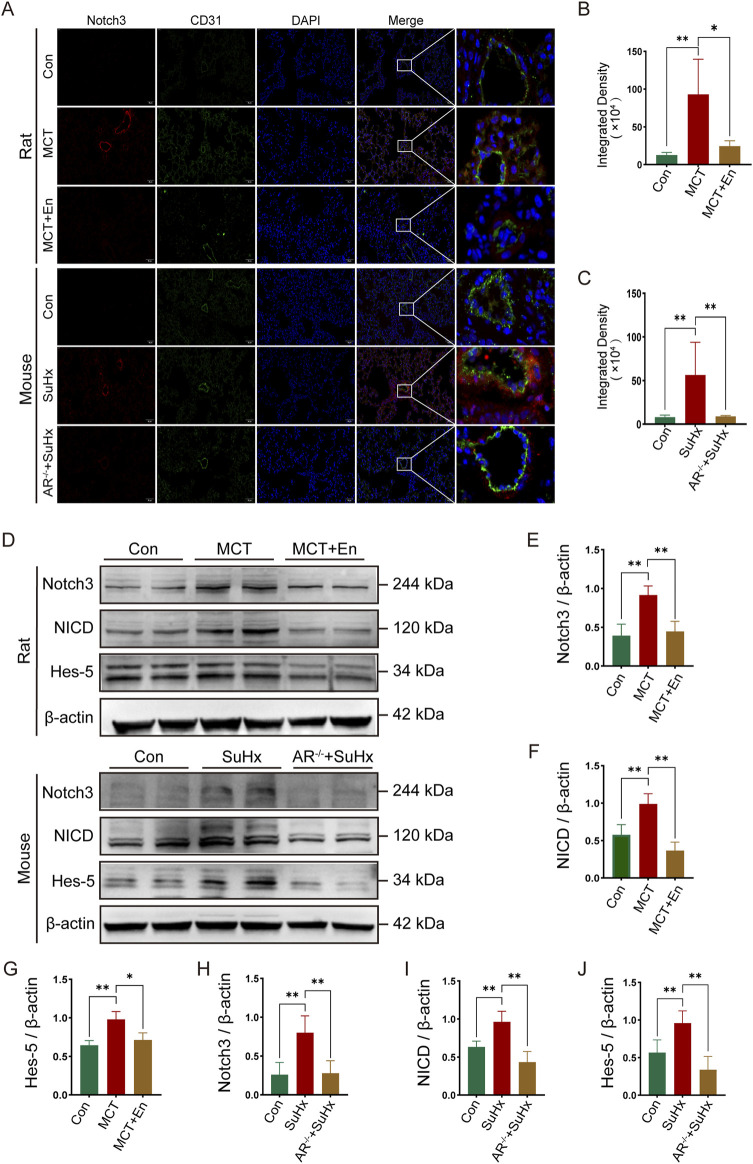
The Notch3/Hes5 signaling pathway is activated in the lung tissues of rats and mice in the model group and is inhibited in rats treated with AR inhibitors and AR^−/−^ mice. **(A)** Notch3 immunofluorescence around pulmonary artery endothelial cells of rats and mice; **(B)** Quantitative statistics of notch3 immunofluorescence in rats and **(C)** in mice; **(D)** Western blot of rat and mouse lung tissues; Quantitative statistics of **(E)** Notch3, **(F)** NICD, and **(G)** Hes-5 proteins in rat lung tissues; Quantitative statistics of **(H)** Notch3, **(I)** NICD, and **(J)** Hes-5 proteins in mouse lung tissues. *p < 0.05, **p < 0.01.

To investigate the Notch3/Hes5 signaling pathway, we performed co-immunofluorescence staining for Notch3 and CD31. The results revealed that Notch3 expression was significantly elevated around the pulmonary arteries in the model groups. In contrast, Notch3 levels were reduced in the pulmonary arteries of MCT-treated rats receiving Enzalutamide and SuHx mice with AR^−/−^(Figures 4A–C). Western blot analysis of lung tissues from rats and mice showed increased levels of Notch3, NICD, and the downstream effector Hes5 in the model groups. However, these protein levels were significantly reduced in the MCT + En and SuHx + AR^−/−^ groups (Figures 4D–J). These findings indicate that the Notch3/Hes5 signaling pathway was inhibited in the MCT + En and SuHx + AR^−/−^ groups.

### 3.5 TFA activation of the Notch3/Hes5 signaling pathway reverses the cardiopulmonary functional improvements in enzalutamide-treated rats and AR−/− mice with PAH

**FIGURE 5:**
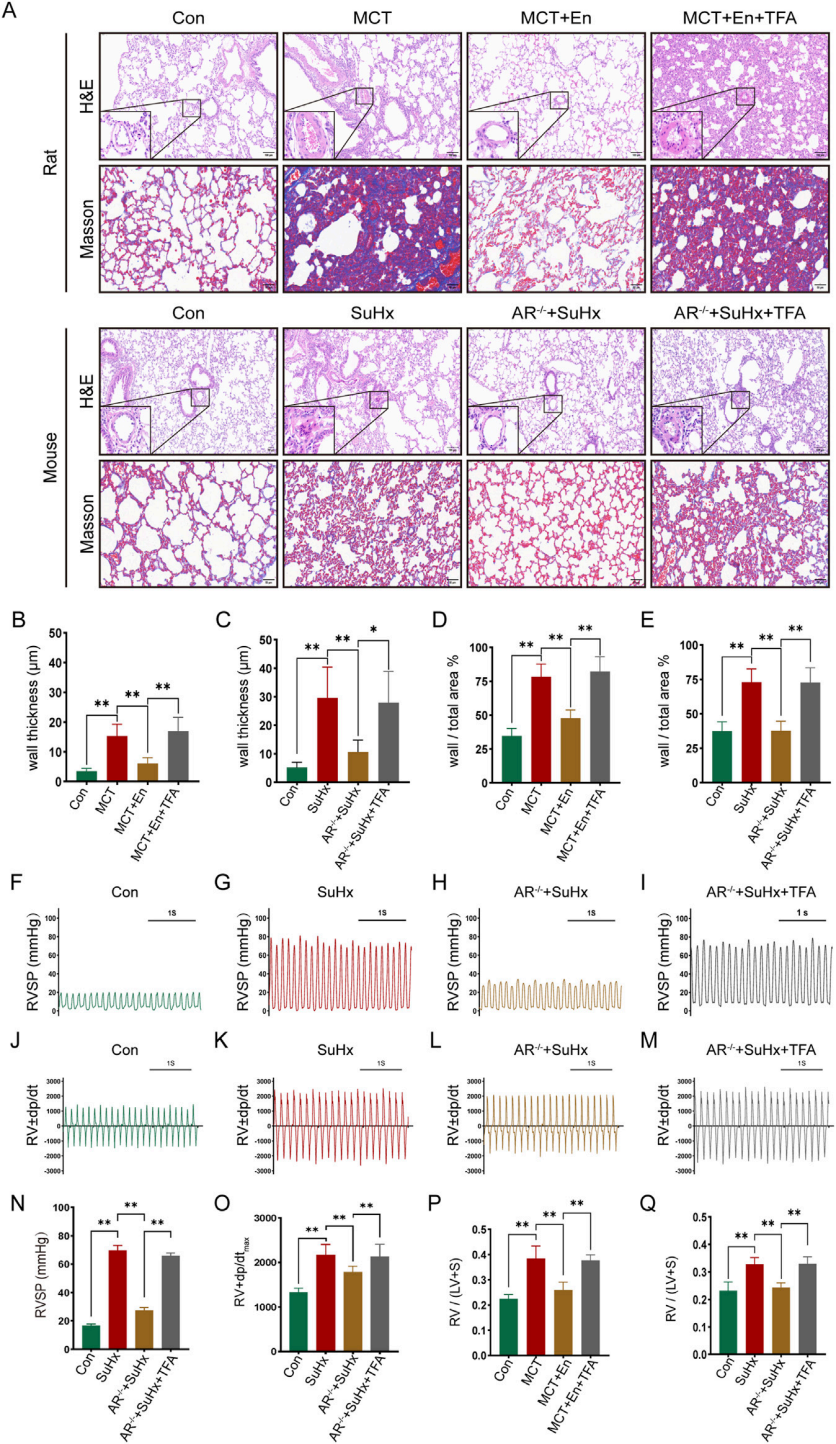
Reversal of cardiopulmonary function in rats and mice after activation of the Notch3/Hes-5 signaling pathway. **(A)** HE staining and Masson staining of rat and mouse lung tissues; **(B)** Statistics of the thickness of small pulmonary artery walls in rats and **(C)** in mice; **(D)** Ratio of arterial wall area to the total arterial lumen area in rats and **(E)** in mice; **(F)** Right ventricular systolic pressure waveforms of normal mice, **(G)** model mice, **(H)** AR^−/−^ mice, and **(I)** AR^−/−^ mice treated with agonists, and **(N)** their corresponding statistical graphs; **(J)** Rate of change of right ventricular systolic pressure in normal mice, **(K)** model mice, **(L)** AR^−/−^ mice, and **(M)** AR^−/−^ mice treated with agonists, and **(O)** statistical graphs of maximum values; **(P)** Right ventricular hypertrophy index in rats and **(Q)** in mice. *p < 0.05, **p < 0.01.

**FIGURE 6:**
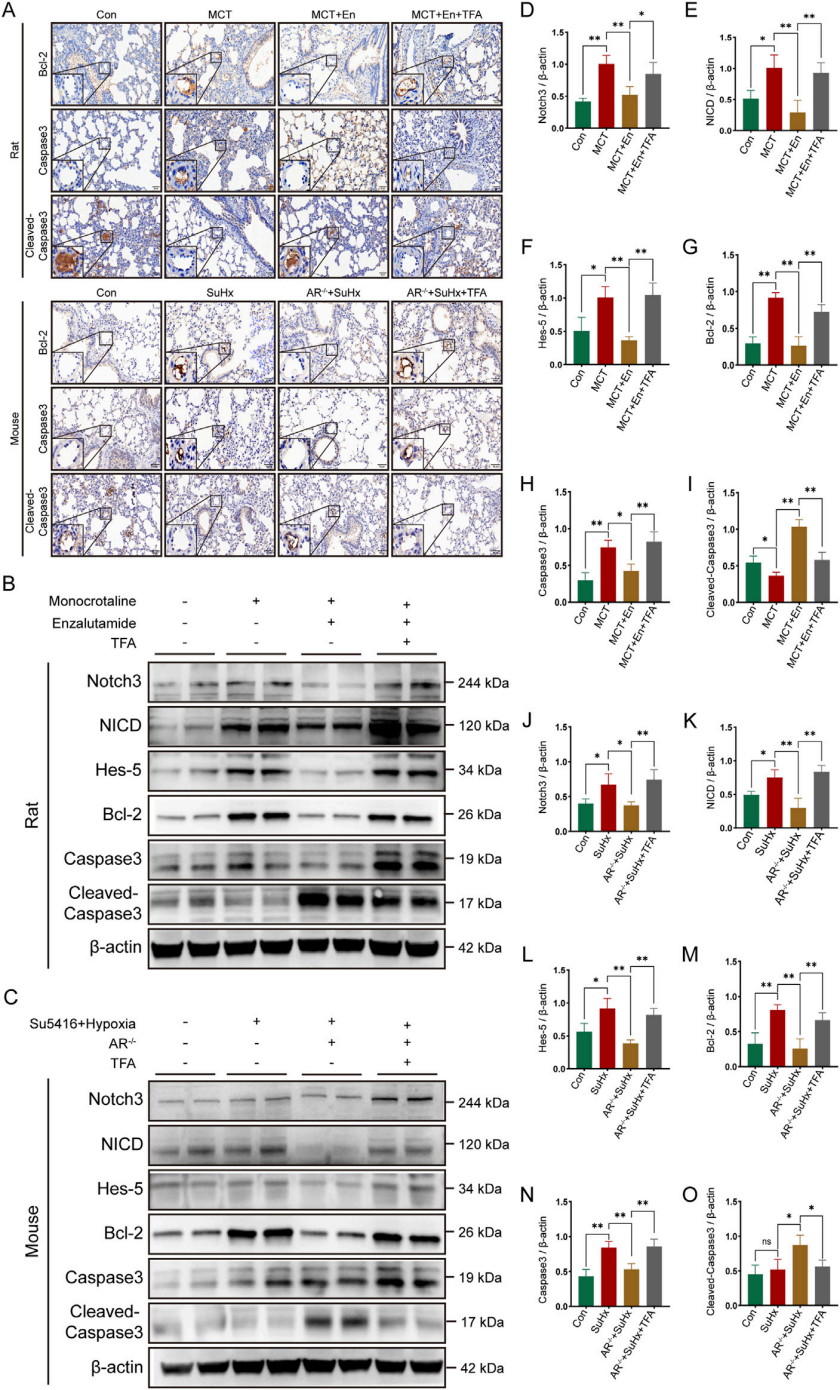
The apoptotic level is decreased in rats and mice after activation of the Notch3/Hes-5 signaling pathway. **(A)** Immunohistochemical staining of Bcl-2, Caspase3, and Cleaved-Caspase3 around the pulmonary artery endothelial cells of rats and mice; **(B)** Western blot of rat lung tissues; **(C)** Western blot of mouse lung tissues; Quantitative analysis and statistics of **(D)** Notch3, **(E)** NICD, **(F)** Hes-5, **(G)** Bcl-2, **(H)** Caspase3, and **(I)** Cleaved-Caspase3 in rat lung tissues; Quantitative analysis and statistics of **(J)** Notch3, **(K)** NICD, **(L)** Hes-5, **(M)** Bcl-2, **(N)** Caspase3, and **(O)** Cleaved-Caspase3 in mouse lung tissues. *p < 0.05, **p < 0.01.

To study the effects of Notch3/Hes5 activation, we used TFA (Jagged-1) to activate the pathway in rats from the MCT + En group and mice from the SuHx + AR^−/−^ group. The animals were divided into four groups (n = 6). Activation of the Notch3/Hes5 signaling pathway reversed the improvements in cardiopulmonary function observed in the MCT + En rats and SuHx + AR^−/−^ mice. Histological analysis revealed that following pathway activation, HE staining showed increased arterial wall thickness, Masson staining indicated exacerbation of pulmonary fibrosis, and the right ventricular hypertrophy index was elevated (Figures 5A–E,P,Q). Additionally, analysis of the right ventricular pressure curves in mice showed increased RVSP and dp/dt_max_ in the SuHx + AR^−/−^ + TFA group (Figures 6F–O).

### 3.6 TFA reverses apoptosis levels by activating the Notch3/Hes5 signaling pathway in enzalutamide-treated rats and AR^−/−^ mice with PAH

Immunohistochemical assays were performed on lung sections from rats and mice across four experimental groups. The expression levels of Bcl-2 and Caspase3, markers associated with Notch3/Hes5 signaling pathway activation, were elevated around the pulmonary arteries, while Cleaved-Caspase3 expression was reduced. These results indicate decreased apoptosis around the pulmonary arteries in rats from the MCT + En + TFA group and mice from the SuHx + AR^−/−^ + TFA group (Figure 6A). Western blot analysis of lung tissue protein levels, including Notch3, NICD, Hes5, Bcl-2, Caspase3, and Cleaved-Caspase3, revealed that activation of the Notch3/Hes5 signaling pathway reversed the therapeutic effects of androgen receptor inhibition. Specifically, TFA treatment led to reduced apoptosis in both MCT + En rats and SuHx + AR^−/−^ mice (Figures 6B–O).

### 3.7 TFA reverses enzalutamide-induced inhibition of the Notch3/Hes5 signaling pathway to modulate apoptosis in hypoxia-exposed PAECs

**FIGURE 7:**
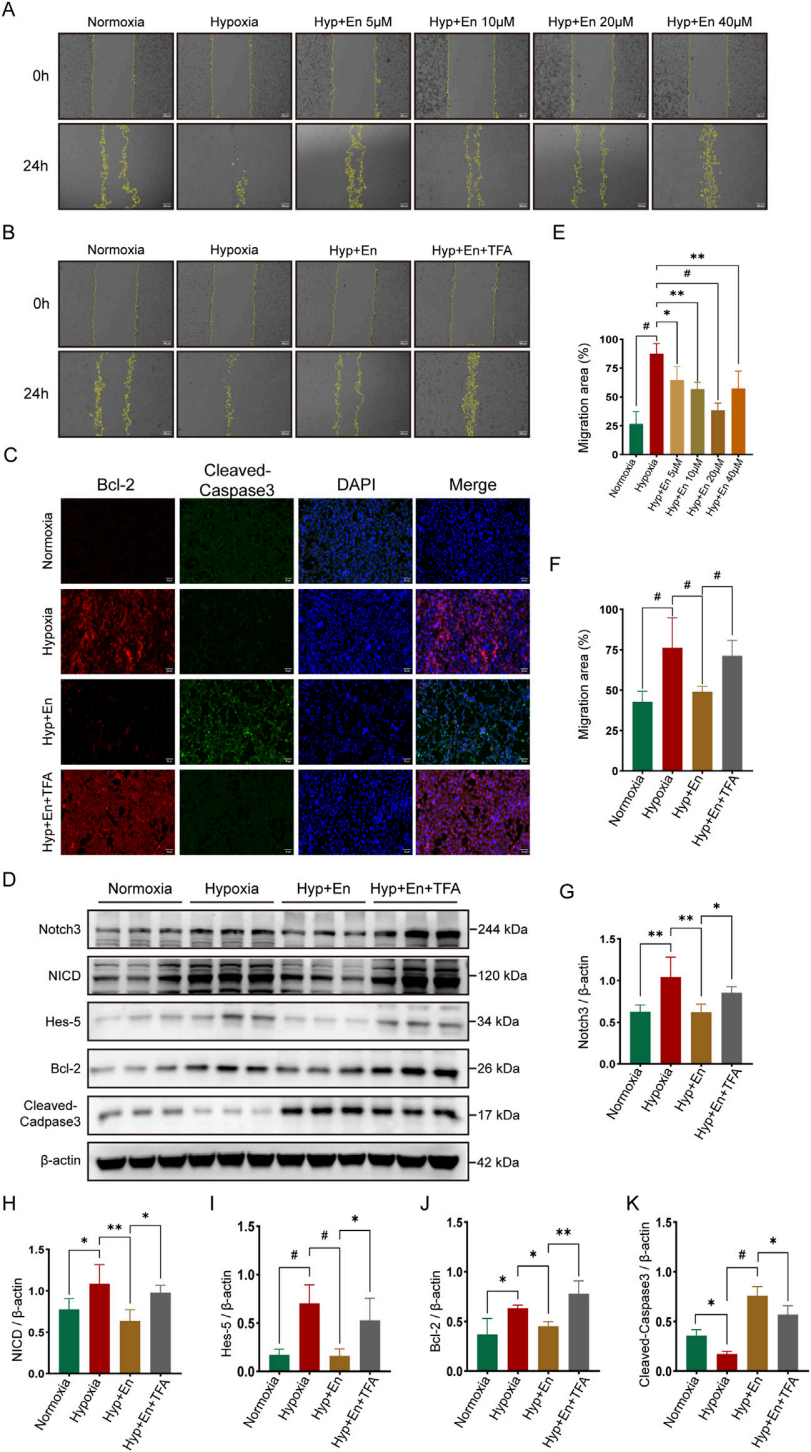
Enzalutamide treatment of PAECs subjected to hypoxia can inhibit the Notch3/Hes5 signaling pathway and increase their apoptotic level, while the apoptotic level is decreased *in vitro* after activation of this pathway. **(A)** Establishment of the enzalutamide dose concentration gradient using a scratch assay and **(E)** statistics of the migration area ratio to determine the optimal treatment concentration; **(B)** Scratch assay of PAECs at 0 h and 24 h and **(F)** statistics of the migration area ratio; **(C)** Immunofluorescence staining of Bcl-2 and Cleaved-Caspase3 in PAECs; **(D)** Western blot detection of proteins in PAECs; Quantitative analysis and statistics of **(G)** Notch3, **(H)** NICD, **(I)** Hes-5, **(J)** Bcl-2, and **(K)** Cleaved-Caspase3 in PAECs. *p < 0.05, **p < 0.01, #p < 0.001.

A cell model of mouse pulmonary arterial hypertension was established by culturing pulmonary artery endothelial cells (PAECs) under 1% hypoxic conditions. The cells were divided into four groups (n = 3): Control (Con), Hypoxia (Hyp), Hypoxia + Enzalutamide (Hyp + En), and Hypoxia + Enzalutamide + TFA (Hyp + En + TFA). A scratch assay was conducted to determine the optimal therapeutic dose of Enzalutamide. PAECs in six-well plates were treated with Enzalutamide at concentrations of 5 μM, 10 μM, 20 μM, and 40 μM. Based on the comparison of scratch assay results at 0 and 24 h, 20 μM was identified as the optimal dose (Figures 7A,E). Subsequent experiments were performed using Enzalutamide at this optimal concentration. Scratch assays were conducted, and the scratched areas at 0 and 24 h were quantified to compare cell migration rates (Figures 7B,F). Immunofluorescence staining for apoptosis markers Bcl-2 and Cleaved-Caspase3 was performed across all groups. Apoptosis was suppressed in the Hyp group, elevated in the Hyp + En group, and reduced again in the Hyp + En + TFA group (Figure 7C). Proteins from the four groups were extracted and analyzed via Western blotting. In the Hyp group, activation of the Notch3/Hes5 signaling pathway was observed, correlating with reduced apoptosis. In the Hyp + En group, Enzalutamide treatment inhibited the Notch3/Hes5 pathway and increased apoptosis levels. However, reactivation of the pathway in the Hyp + En + TFA group was associated with a reduction in apoptosis (Figures 7D,G–K).

## 4 Discussion

Currently, the treatment of pulmonary arterial hypertension (PAH) primarily involves supportive therapies, such as thrombus prevention and the use of pulmonary vasodilators (Shah et al., 2023; Lau et al., 2017; Boucly et al., 2021; Spiekerkoetter et al., 2019). Lung transplantation remains the only potentially curative treatment for PAH, though its applicability is limited (Chin et al., 2024; Kolaitis, 2023). A review of the literature reveals significant gender differences in the prognosis of PAH, with males often experiencing more severe disease progression (Takase et al., 2021). Furthermore, androgen receptors are widely expressed in blood vessels and play a crucial role in vascular remodeling (Abi-Ghanem et al., 2020; Takov et al., 2018; Yoshida et al., 2016; Ikeda et al., 2009). These observations led us to hypothesize that androgen receptors may contribute to the pathophysiology of PAH and the gender disparities observed in disease prognosis (Cheron et al., 2021; Foderaro and Ventetuolo, 2016).

Apoptosis is critical in the formation and progression of PAH (Yung et al., 2020). Enzalutamide, an androgen receptor inhibitor, exhibits a favorable pro-apoptotic effect in diseases such as prostate cancer (Scher et al., 2012). To explore the role of androgen receptors in PAH, we administered Enzalutamide via intraperitoneal injection in MCT-induced rats to inhibit androgen receptor expression and used gene editing technology to knock out androgen receptors in C57BL/J6 mice. Both treatments resulted in improved cardiopulmonary function. We then measured Bcl-2, Caspase3, and Cleaved-Caspase3 levels in lung tissues and found increased apoptosis. Bcl-2 primarily exerts anti-apoptotic effects by interacting with pro-apoptotic proteins, preventing the initiation of programmed cell death. Caspase3 is a key protease in the execution phase of apoptosis, and Cleaved-Caspase3 promotes apoptosis by cleaving various cellular substrate proteins, leading to cell structural damage. These findings suggest that inhibiting androgen receptors may reduce the proliferation of arterial endothelial cells in PAH by enhancing apoptosis in lung tissues.

The Notch3/Hes5 signaling pathway plays a crucial role in the pathogenesis of PAH by regulating key cellular processes such as proliferation, apoptosis, and vascular remodeling (Bodas et al., 2022; Pokharel et al., 2023; Winicki et al., 2024). In various cancer models, this pathway has been shown to modulate cell proliferation and apoptosis (Katoh and Katoh, 2020; Yamaguchi et al., 2008; Brahim et al., 2023; Liu et al., 2022; Somnay et al., 2017). Furthermore, the Notch3/Hes5 axis is essential for regulating endothelial and smooth muscle cell functions, as well as maintaining the structural and functional stability of blood vessels (Gaengel et al., 2009). To explore the potential interaction between androgen receptors and the Notch3/Hes5 pathway in PAH, we assessed the expression levels of Notch3, NICD, and Hes5 in lung tissues. Our results revealed that the Notch3/Hes5 pathway was activated in the PAH group, with activation suppressed following androgen receptor inhibition. These findings suggest that the therapeutic effects of androgen receptor blockade may involve modulation of the Notch3/Hes5 signaling pathway, ultimately increasing apoptosis in pulmonary tissues.

**FIGURE 8:**
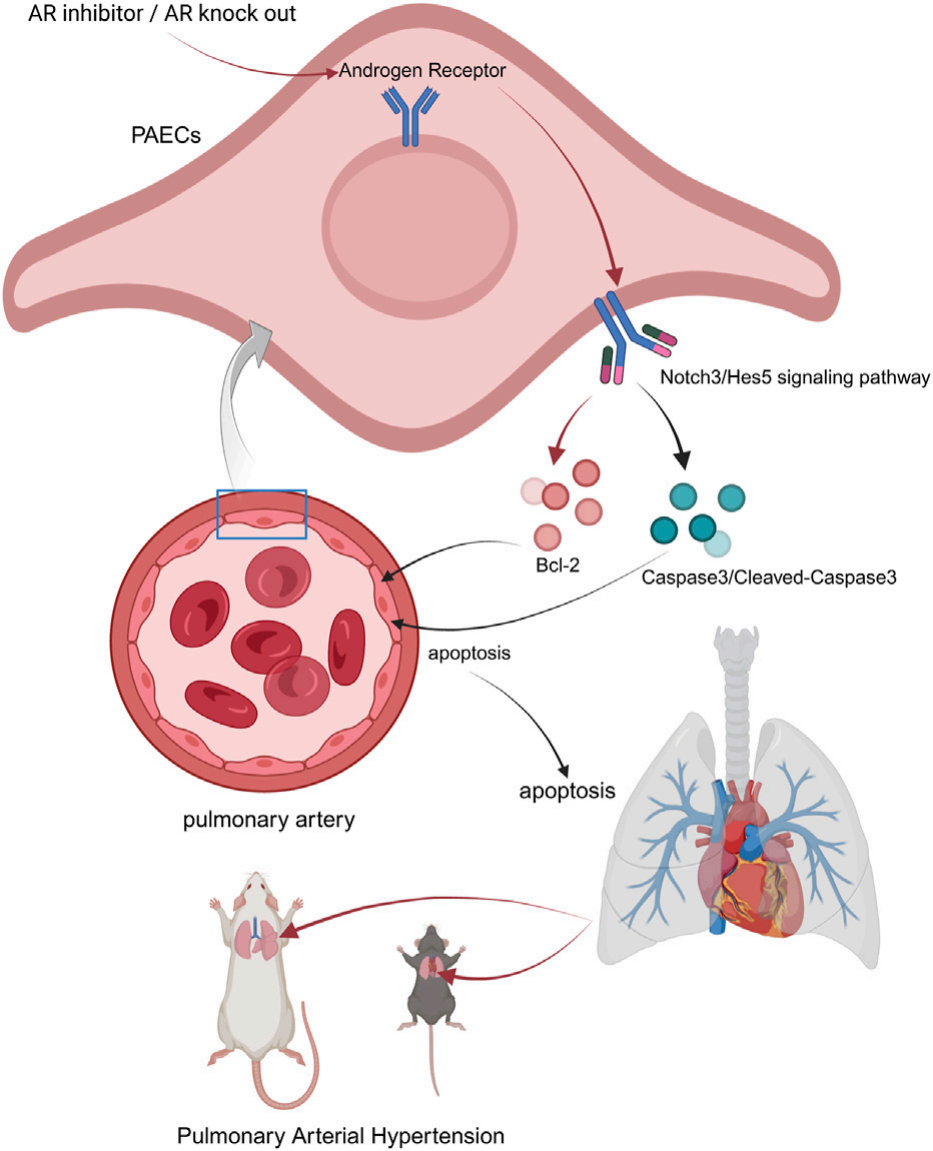
Possible underlying mechanism of AR inhibitors in the treatment of PAH. Androgen receptor inhibitors inhibit the Notch3/Hes5 signaling pathway by suppressing androgen receptors within pulmonary artery endothelial cells. Some apoptosis-related molecules are regulated by this pathway, jointly upregulating the apoptosis level of PAECs, thereby resisting pathological changes such as the proliferation of PAECs caused by PAH (created in https://BioRender.com).

To further test this hypothesis, we used TFA (Jagged-1), a Notch pathway agonist, to activate the Notch3/Hes5 signaling pathway in PAH rats and mice. Notch3/Hes5 activation exacerbated cardiopulmonary dysfunction and decreased apoptosis in lung tissues. At the cellular level, Enzalutamide treatment in the PAH model led to increased apoptosis in pulmonary artery endothelial cells, whereas activation of the Notch3/Hes5 pathway reduced apoptosis. These results suggest that androgen receptor inhibitors may exert therapeutic effects in PAH by inhibiting the Notch3/Hes5 pathway, thereby increasing apoptosis in pulmonary tissues and alleviating disease progression (Figure 8) (Jurasz et al., 2010).

Our previous studies examined the relationship between the calcium-sensing receptor (CaSR) and pulmonary arterial hypertension (PAH) (Guo et al., 2017). Bcl-2 family proteins are important regulators of calcium channels in the endoplasmic reticulum and mitochondria (D'Orsi et al., 2017; Pinton and Rizzuto, 2006; He et al., 1997). Given the critical role of CaSR in PAH, further investigation is needed to elucidate the direct association between the Bcl-2 family and PAH (Wang et al., 2021). The Wnt/β-catenin signaling pathway plays a significant role in the development and progression of pulmonary hypertension associated with pulmonary fibrosis (Lin et al., 2024). This pathway facilitates the transformation of alveolar epithelial cells into interstitial cells, thereby contributing to the onset of pulmonary hypertension. Pulmonary fibrosis of varying degrees frequently coexists with PAH (Fulton et al., 2019). Thus, it is essential to explore whether the androgen receptor is involved in epithelial-mesenchymal transition (EMT) and endothelial-mesenchymal transition (EndMT), which may further contribute to the pathogenesis of PAH (Jiang et al., 2019; Li et al., 2022; Gorelova et al., 2021; Zheng et al., 2021; Shu et al., 2024; Yao et al., 2024).

This study is the first to investigate the role of gender differences in the treatment of PAH, with a specific focus on how androgen receptors influence disease progression. Through a series of experiments, we have demonstrated that androgen receptors regulate apoptosis levels in pulmonary artery endothelial cells via the Notch3/Hes-5 signaling pathway. These findings provide important insights into the potential use of androgen receptor inhibitors as a novel therapeutic option for PAH. However, the precise molecular mechanisms underlying the effect of androgen receptors on PAH remain incompletely understood. This study is not without limitations. One notable drawback is the omission of female animal models. Moving forward, we are committed to refining this experiment and delving deeper into this area of research. Therefore, future research should focus on more detailed investigations into the interactions between androgen receptors and the Notch3/Hes5 pathway, as well as other potential signaling mechanisms, to refine our understanding and optimize therapeutic strategies for PAH.

## Data Availability

The original contributions presented in the study are included in the article/Supplementary Material, further inquiries can be directed to the corresponding author.
